# Primary intraosseous squamous cell carcinoma arising from an odontogenic keratocyst: case series and literature review

**DOI:** 10.4317/medoral.23947

**Published:** 2020-10-09

**Authors:** Peng Ye, Tai Wei, Yan Gao, Wen-Bo Zhang, Xin Peng

**Affiliations:** 1 Department of Stomatology, Beijing Hospital, National Center of Gerontology, Institute of Geriatric Medicine, Chinese Academy of Medical Sciences, Beijing, China; 2 Peking University School and Hospital of Stomatology First Clinical Division, Beijing, China; 3 Department of Oral Pathology, Peking University School and Hospital of Stomatology, Beijing, China; 4 Department of Oral and Maxillofacial Surgery, Peking University School and Hospital of Stomatology, Beijing, China

## Abstract

**Background:**

The aim of this study was to investigate the clinicopathologic features of primary intraosseous squamous cell carcinoma arising from an odontogenic keratocyst (PIOSCC ex OKC) and comprehensively improve the understanding of this disease.

**Material and Methods:**

We retrospectively investigated five cases of PIOSCC ex OKC at Peking University School and Hospital of Stomatology. We also conducted a systematic review of studies on PIOSCC ex OKC by using online databases from their inception until February 2020.

**Results:**

In our series of five cases, all lesions were located in the mandible. Three cases (60%) showed recurrent OKCs and two cases (40%) showed primary OKCs. During the follow-up period, one patient died of local relapse. No patients developed metastasis. On the basis of our literature survey, we selected 22 articles reporting 29 patients with PIOSCC ex OKC. Seven of these patients (24.1%) showed local recurrence, three patients (10.3%) developed cervical metastasis, three patients (10.3%) developed distant metastasis (in the pleura in one case and in the lung in two cases), and seven patients died from the disease during the follow-up period. The disease-specific 5-year survival rate in the study group was 53.2%. Through univariate and multivariate analysis, local recurrence was identified as the only significant independent prognostic factor for survival (*P* < 0.05).

**Conclusions:**

The results suggest that PIOSCC ex OKC is a rare intermediate-grade malignancy. Although elective neck dissection is typically unnecessary, adequate therapy should be applied to achieve the lowest local recurrence rate possible to ensure a favorable survival rate.

** Key words:**Primary intraosseous squamous cell carcinoma, odontogenic keratocyst, prognosis.

## Introduction

In the World Health Organization (WHO) classification of 2017, odontogenic keratocyst (OKC) is re-termed as a benign intraosseous cyst of odontogenic origin with local aggressive and infiltrative potential (WHO Classification of Head and Neck Tumours, 4th ed). Squamous cell carcinoma (SCC) has the potential to develop in the lining of OKCs. In accordance with the latest WHO classification, this malignancy is defined as a squamous cell carcinoma arising within the jaws without connection to the oral mucosa in the presence of a keratocystic odontogenic tumour. Although primary intraosseous squamous cell carcinoma (PIOSCC) arising from odontogenic cysts has been reported occasionally, studies investigating the clinicopathologic features of PIOSCC arising from OKC (PIOSCC ex OKC) are extremely rare, because of which these entities remain poorly understood (1). Therefore, the aim of the present study was to retrospectively analyze the clinicopathologic characteristics of five cases of PIOSCC ex OKC and systematically review the literature to improve the understanding of this disease.

## Material and Methods

- Case series and characteristics

The medical and pathological records of all patients treated for PIOSCC ex OKC at the Department of Oral and Maxillofacial Surgery at the Peking University School and Hospital of Stomatology from 2000 through 2019 were retrospectively reviewed. Five cases of PIOSCC ex OKC were selected for this study. Demographic data, tumor site, T stage, clinical manifestation, treatment, pathological diagnosis, and follow-up information were obtained (1). The mean follow-up time was 55.8 months (range, 20 to 128 months).

- Literature review

We searched the Cochrane Library, Wiley Online databases, EMBASE, PubMed, and China National Knowledge Infrastructure (CNKI) without language or date restrictions. The following search algorithm was included: “primary intraosseous squamous cell carcinoma” or “squamous cell carcinoma” in combination with “keratocystic odontogenic tumor” or “odontogenic keratocyst.” All articles that reported on PIOSCC ex OKC and published in peer-reviewed journals were included after being comprehensively reviewed. Studies without a definite diagnosis or therapeutic and follow-up information were excluded. And in accordance with the updated WHO classification of 2017, studies reporting PIOSCC arising from orthokeratinised OKC (which is newly categorized as orthokeratinised odontogenic cyst) were excluded. The following details were recorded for all eligible studies: author names, year of publication, clinical manifestation, pathologic diagnosis, treatment modality, therapeutic outcomes, and duration of follow-up. The mean follow-up time was 23.1 months (range, 3 to 72 months).

- Statistical analysis

All statistical analyses were performed using SPSS 20.0 (SPSS, Inc, Chicago, IL). Common clinicopathologic factors (ie, age, gender, lesion location and origin, malignant type, therapeutic option, local recurrence, cervical metastasis and distant metastasis) were entered into a Cox proportional hazard model in univariate and multivariate analysis in order to find the independent prognostic factors. Disease-specific survival was analyzed with the Kaplan-Meier method, and the log-rank test was applied to identify intergroup survival differences. Hazard ratios and 95% confidence intervals for multivariate models were computed with the use of Cox proportional hazard regression models (forward stepwise method). *P* < 0.05 was considered statistically significant.

## Results

- Patients' clinicopathologic characteristics

**Figure 1:**
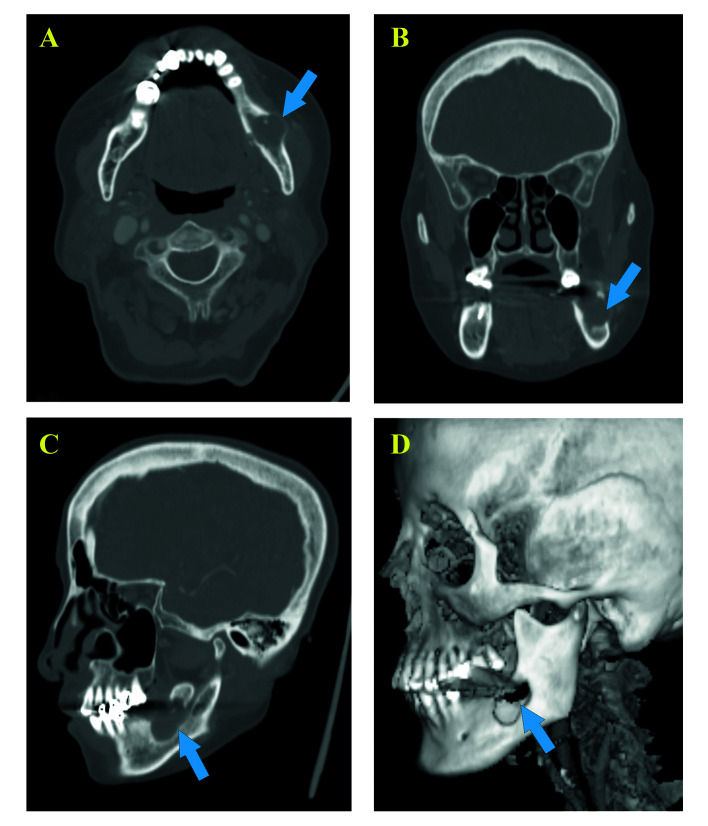
Spiral computed tomography (SCT) findings of PIOSCC ex OKC in the left body of the mandible. (A) Axial plane view showing local lysis of lingual cortex and trabeculae, lytic expansion of buccal cortex. (B, C) Coronal and Sagittal plane views showing local osteolysis and involvement of mandibular canal wall. (D) Three-dimensional image showing an osteolytic lesion with relatively irregular boundaries in the left molar area of the mandible.

The five patients with PIOSCC ex OKC included three men and two women with a mean age of 48.2 years (range, 35 to 71 years). All lesions were located in the mandible (Fig. 1).

**Figure 2:**
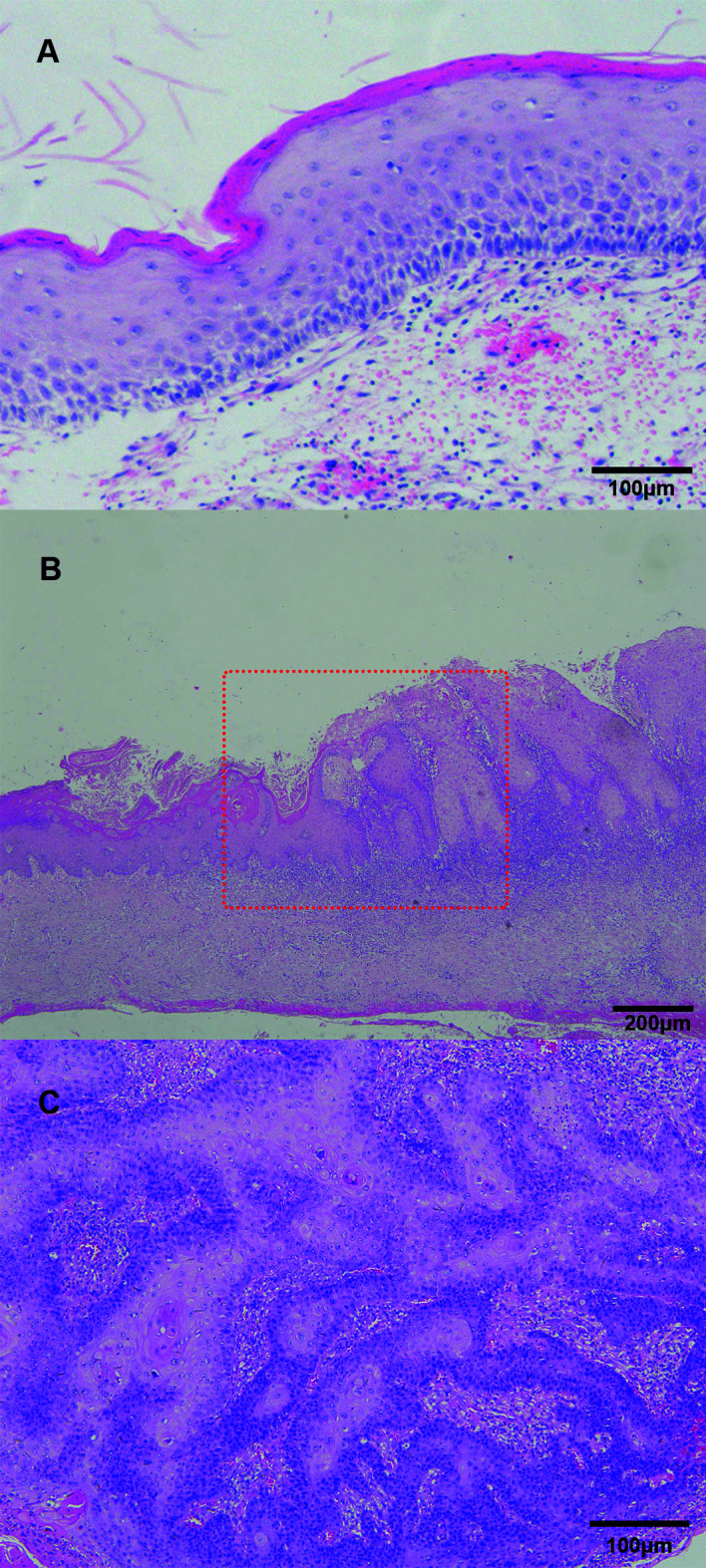
Photomicrographs of surgical specimen (H&E staining). (A) The cyst wall lined by a parakeratinized epithelium without rete ridges and the basal layer lined by palisaded tall columnar cells (magnification, ×10; bar = 100μm). (B). Transition zone (the dotted box) from benign cyst lesion (the left part) to dysplasia and early infiltrative carcinoma (the right part) (magnification, ×4; bar = 200μm). (C) Well-differentiated squamous cell carcinoma arising from the dysplastic cyst epithelium (magnification, ×10; bar = 100μm).

Three cases (60%) showed recurrent OKCs and two cases (40%) showed primary OKCs. The mean medical course was 5 years before the diagnosis of PIOSCC. Microscopic examination revealed the parakeratinised stratified epithelial lining of OKCs in all five cases. Malignancy was classified as an invasive well-differentiated SCC in four cases (80%) and carcinoma-in-situ in one case (20%) (Fig. 2). Two cases were graded as T3, two cases as T4a, and one case as T2. The clinical complaints reported by the patients included local pain (n = 4), swelling (n = 5), and numbness (n = 2). All patients underwent extended excision of lesions, and three patients underwent selective neck dissection and postoperative radiotherapy additionally. No positive lymph node was found in any neck dissection specimen. During the follow-up period, two patients (40%) developed local relapse and one patient died of uncontrollable local recurrence. No patient developed metastasis. The patients' clinicopathologic data are presented in Table 1.

- Characteristics of the eligible studies

In total, 22 articles reporting 29 cases of PIOSCC ex OKC were selected for systematic analysis based on our inclusion and exclusion criteria (2-21; Zhang J *et al*. 2013 and Liu C *et al*. 2017 published in chinese). Of these articles, 19 were single case reports and three were case series. All 22 reports were retrospective studies. Detailed characteristics of the eligible studies are summarized in Table 2. The 29 patients with PIOSCC ex OKC included 17 men (58.6%), 11 women (37.9%), and one case unspecified with a mean age of 48.8 years (range, 15 to 81 years). 19 cases (65.5%) involved tumors anatomically derived from the mandible and nine cases (31.0%) involved tumors occurring in the maxilla while the details in one case were unspecified. Pathologically, PIOSCCs originated in recurrent OKCs in 11 cases (37.9%), 16 cases (55.2%) had associated primary OKCs, and no information was mentioned for two cases. The malignancy in 28 cases (96.6%) was identified as SCC and verrucous carcinoma in one case (3.4%). Most patients (16 of 29, 55.2%) underwent extensive resection alone; three patients (10.3%) underwent simple enucleation alone; seven patients (24.1%) received extensive surgery followed by adjuvant radiotherapy; two patients (6.9%) received chemoradiotherapy after extensive surgery and one patient (3.4%) received extensive resection after preoperative chemoradiotherapy. In total, during the follow-up period, seven patients (24.1%) developed local recurrence, three patients (10.3%) showed cervical metastasis, three patients (10.3%) developed distant metastasis (one in the pleura, two in the lung), and seven patients (24.1%) succumbed to this disease entity.

**Figure 3:**
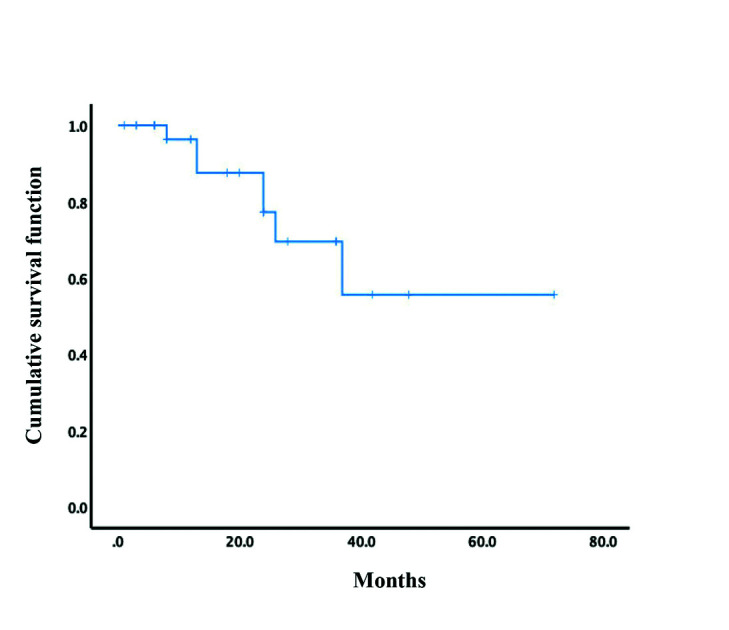
Cumulative survival curve of patients with PIOSCC ex OKC in the literature.

The disease-specific 5-year survival rate in the study group was 53.2%. The disease-specific survival curve is showed in Fig. 3. Univariate and multivariate disease-specific survival analyses of the included 29 patients are listed in Table 3. In the univariate analysis, PIOSCC derived from recurrent OKC (*P* = 0.032), surgical excision without radiotherapy (*P* = 0.036) and local recurrence (*P* = 0.002) were identified as significant predictive factors of poor survival; nevertheless, only local recurrence turned out to be a significant independent negative determinant of survival (*P* = 0.006) in the multivariate analysis.

**Table 1:**
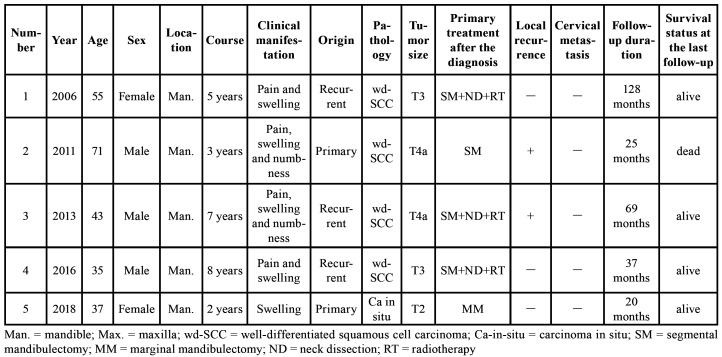
Patients with squamous cell carcinoma arising from an OKC.

**Table 2:**
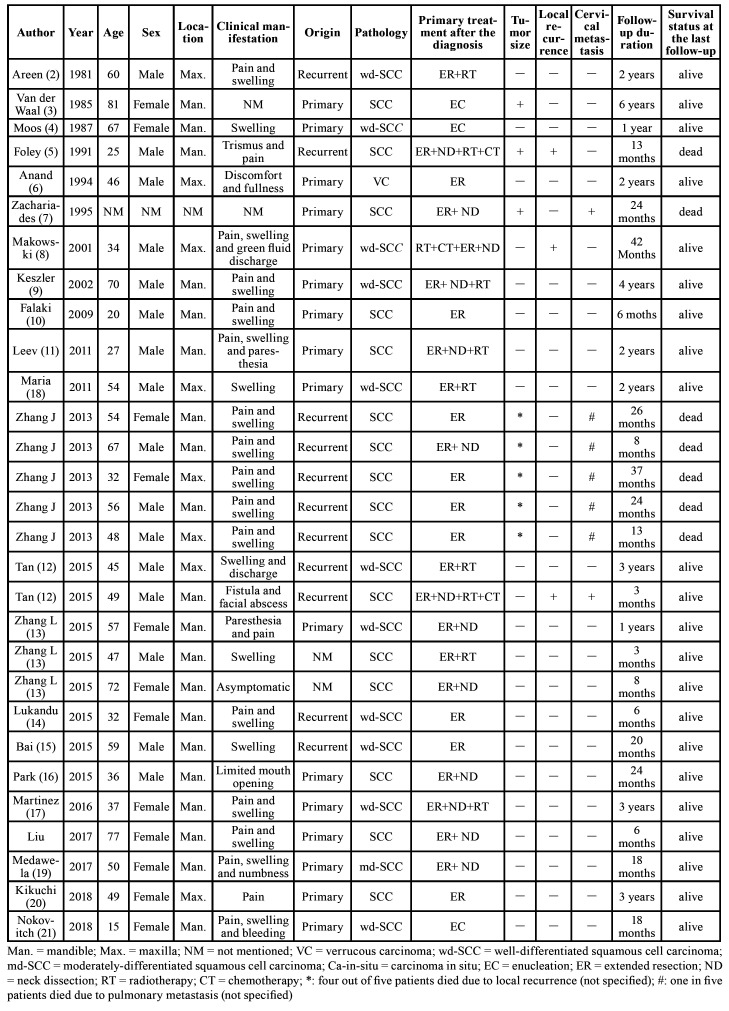
Patients with squamous cell carcinoma arising from an OKC reported from 1981 to 2019.

**Table 3:**
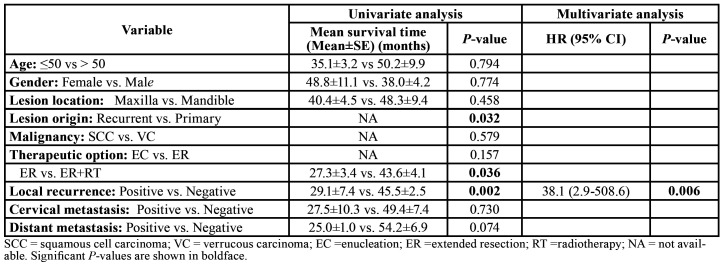
Univariate and multivariate disease-specific survival analyses of patients in literature.

## Discussion

To date, the pathogenesis of PIOSCC ex OKC and the malignant transformation rates of OKC are still unknown. One of the widely accepted hypotheses is that the longstanding inflammatory microenvironment acts as a key factor for malignant degeneration of the epithelium of OKC (22).

A variety of patients with PIOSCC ex OKC present with an uneventful cyst or swelling for many years before new symptoms such as local pain, facial paresthesia or numbness, and rapid growth occur (12-15). This phenomenon is in agreement with the mean clinical course of 5 years before diagnosis in our series. The long historical course could result in persistence or even deterioration of the chronic inflammation of the epithelial lining. In addition, due to its infiltrative clinical biological behavior, the local recurrence rate of OKCs was reported up to 62% after surgical treatment (22). Multiple relapses not only accelerate the inflammatory process but also increase the malignant potential of the epithelium lining. Chronic inflammation is featured by persistent tissue damage and damage-induced cellular proliferation. Cell proliferation is usually associated with "dysplasia" which is regarded as the precursor of carcinoma. Moreover, macrophages and T-lymphocytes in the inflammatory microenvironment may release tumor necrosis factor-alpha and macrophage migration inhibitory factor to aggravate DNA damage (23). In our series, three out of five lesions (60%) originated from recurrent OKC. Similarly, 11 cases (37.9%) of PIOSCC ex OKC in the systematic review were derived from recurrent OKC. Theoretically, the frequent recurrence of OKC is related with multiple factors, such as treatment modalities employed, operative experience, size and location of the lesion, and existence of nevoid basal cell carcinoma syndrome, etc. Therefore, if OKC is properly treated and recurs, histomorphological and sometimes immunohistochemical characteristics of the complete OKC specimen should be exhaustively examined by pathologist to rule out the possible pre-malignant or malignant transformation of recurrent (particularly multiple or with long historical course) OKCs (17). Overall, timely and radical removal of OKCs could decrease the incidence of PIOSCC ex OKC.

The lesions in all cases in our series and most of cases (65.5%) in the present literature review were located in mandible, which is consistent with the predilection site of OKCs (13). The main clinical manifestations in all the patients in our series and review were swelling and pain, due to the increasing accumulation of cystic fluid and local inflammatory reaction within the lesion. Notably, two cases in our series and three patients in the review presented with numbness or paresthesia, which was possibly on account of the invasion of the inferior alveolar nerves by malignancy. Hence, once paresthesia or even numbness is encountered in an odontogenic cystic lesion, malignant degeneration should be highly suspected.

Earlier investigations reported that OKC is predominantly parakeratotic, but occasionally orthokeratotic (22). However, in the updated WHO classification of 2017, Orthokeratotic OKC is categorized as orthokeratinised odontogenic cyst as its less infiltrative biological behavior and lower recurrence after treatment. Hence, the pathological slides of all our five patients were reexamined to ensure consistence with the new classification. Whilst, it is noteworthy that five cases of PIOSCC deriving from orthokeratinised OKC were reported in our literature survey. All these patients were disease free postoperatively at the last follow-up. It is indicative that orthokeratinised epithelial lining also has malignant potential and orthokeratinisation seems not always a favorable variable for odontogenic cysts (24-27).

In the present literature review, the rates of cervical and distant metastasis were both 10.3%. In contrast, the cervical metastasis rate of all PIOSCCs arising from jawbones was reported to range from 36.5% up to 70.1% (28,29), which was much higher than our finding. This suggests that selective neck dissection and chemotherapy are not supposed to be planned after comprehensive examination unless metastasis is identified clinically before surgery. Local recurrence was found in 40% and 24.1% of the cases in our present series and literature review respectively. Hence, extensive *en bloc* resection is strongly recommended and subsequent radiotherapy ought to be applied in case of positive surgical margin to decrease relapse.

To date, few studies have comprehensively evaluated the predictive and prognostic factors for the survival of patients with PIOSCC ex OKC due to the fairly low incidence. The overall disease-specific 5-year survival rate of PIOSCC ex OKC in this review was 53.2%. In contrast, the estimated 5-year overall survival of de novo PIOSCC and those derived from other cystic lesions range from 36.3% to 38.8% (29,30). This discrepancy suggests that PIOSCC ex OKC can be classified as an intermediate-grade malignancy associated with better prognosis with respect to the survival rate.

Through univariate and multivariate analysis, local recurrence was identified as the only significant independent prognostic factor for survival (*P* < 0.05). Clinicopathologic features, including age, gender, anatomic location, lesion origin, malignant type, treatment modalities, cervical and distant metastasis were also analyzed, but no significant relationship with survival was observed. Although treatment modalities statistically showed no significant effect on survival in our analysis, the well-acknowledged approach to decrease local recurrence is focal extended resection and adjuvant radiotherapy in case of positive surgical margin. Hence, radical resection and post-operative radiotherapy when necessary are recommended to improve the survival of patients with PIOSCC ex OKC. Moreover, these findings warrant future large-scale investigation.

## Conclusions

This study confirmed that PIOSCC ex OKC is a rare intermediate-grade malignancy. The authors believe that timely and complete removal of OKCs could decrease the incidence of PIOSCC ex OKC. Although elective neck dissection is usually unnecessary, adequate therapy should be applied to achieve the lowest local recurrence ensuring a favorable survival rate.
